# A new species of *Bothriembryon* (Mollusca, Gastropoda, Bothriembryontidae) from south-eastern Western Australia

**DOI:** 10.3897/zookeys.581.8044

**Published:** 2016-04-14

**Authors:** Corey S. Whisson, Abraham S.H. Breure

**Affiliations:** 1 Western Australian Museum, Locked Bag 49, Welshpool, WA 6106, Australia; 2 School of Veterinary and Life Sciences, Murdoch University, Murdoch, WA 6150, Australia; 3 Naturalis Biodiversity Center, P.O. Box 9517, 2300 RA Leiden, the Netherlands; 4 Royal Belgian Institute of Natural Sciences, Vautierstraat 29, Brussels, Belgium

**Keywords:** Western Australia, Orthalicoidea, ecology, anatomy, micro-CT

## Abstract

*Bothriembryon sophiarum* **sp. n.** is described, based on shell and anatomical morphology, from the coastal area of south-easternmost Western Australia. This is the first description of a new extant Australian bothriembryontid in 33 years. The shell of *Bothriembryon sophiarum* is slender with a unique teleoconch sculpture. It is found in low coastal scrub on cliff edges and escarpments and because of its restricted distribution, qualifies as a short range endemic.

## Introduction

Along with the diverse and generally more northern and eastern Camaenidae, the endemic Australian genus *Bothriembryon* (Bothriembryontidae) forms a large and characteristic component of the Australian terrestrial molluscan fauna, particularly in Western Australia (Iredale 1939; Kershaw 1985; Solem 1998). Thirty five extant and seven fossil *Bothriembryon* species are currently known from Australia (Iredale 1939; Breure 1979; Smith 1992; Richardson 1995; Smith et al. 2002; Breure and Whisson 2012) but many undescribed species have been proposed based on specimen identifications by former Western Australian Museum malacologists.

Recent taxonomic work on this group has been limited, with the last description of a new extant species being over thirty years ago (Hill et al. 1983). The majority of *Bothriembryon* species are limited to mesic south-western Western Australia with two species endemic to South Australia, one species to the lower Northern Territory and one species to south-eastern Tasmania. During the 1970’s a somewhat slender shell from the Caiguna and Cocklebiddy areas was identified as a new species by Western Australian Museum malacologist Hillary Merrifield but was never named. This taxon is described herein.

## Material and methods

A total of 22 lots comprising 242 specimens were examined from the malacological collection of the Western Australian Museum. Three relaxed formalin-fixed specimens were used for 3D visualisation (Walker et al. 2014) and stained in a solution of 1% iodine in 70% ethanol for four days. Due to the narrowly elongated shape of the shells the staining of the upper whorls is less strong than those of the animal extending outside the shell (Fig. 5A). Subsequently they were scanned using the Nikon Metrology HMX ST 225 micro-CT scanner at the Imaging and Analysis Centre at the Natural History Museum, London. This system is equipped with a detector panel (2000 × 2000 pixels) with a maximum resolution of 5 μm and a maximum energy of 225 kV. A tungsten reflection target was used with the following parameters: 180 kV, 180 μA, 500 ms exposure time and 3,142 projections were taken. Images acquired during the scanning process were subsequently reconstructed using CT Pro (Nikon Metrology, Tring, UK), which employs a modified version of the back-projection algorithm created by Feldkamp et al. (1984). Output files were analysed with ImageJ 2.0.0-rc-9 (2D), and Avizo 8.1 and Mimics 15.01 (3D).

Shell dimensions followed the methods figured by Breure (1974: fig. 2) and Kendrick and Wilson (1975: fig. 1) for whorl counts. Measurements were made through digital calipers to 0.1 mm for maximum shell height (H) and maximum shell diameter (D) and a Leica M80 Dissecting Microscope for number of shell whorls (W), number of protoconch whorls (P) and number of spiral lines on the penultimate whorl (SP). Finer measurements of height of aperture (HA), width of aperture (WA) and height of last whorl (LW) were taken from digital photographs using a Leica MZ16A microscope with Leica DFC500 camera. Photographs of live and preserved specimens were also taken with this equipment. Anatomical features are following the terminology of Tompa (1984), and—contrary to Breure (1978)—proximal and distal refer to organ (or parts of organ) positions relative to the direction of the gamete flow, i.e. from tip of flagellum (proximal) to genital pore (distal).

Abbreviations of depositories: AM, Australian Museum, Sydney, Australia; RMNH, Naturalis Biodiversity Center, Leiden, the Netherlands; WAM, Western Australian Museum, Perth, Australia. Data for material examined have been transcribed as per specimen labels and distributional maps were plotted using ArcGis 10.1 software.

## Systematics

### Superfamily Orthalicoidea Albers, 1860 Family Bothriembryontidae Iredale, 1937 Subfamily Bothriembryontinae Iredale, 1937 Genus *Bothriembryon* Pilsbry, 1894

#### Subgenus Bothriembryon

Taxon classificationAnimaliaStylommatophoraBothriembryontidae

Pilsbry, 1894

##### Type species.

*Helix melo* Quoy & Gaimard, 1832 by original designation

##### Remarks.

The use of subgenera within this genus is disputed. Breure (1978, 1979) recognized Bothriembryon (Bothriembryon) and Bothriembryon (Tasmanembryon) while Kershaw (1986), after a detailed study of external and internal morphology, concluded that his evidence suggested only one generic unit. We follow the opinion of Breure (1978, 1979) supported in recent reviews (Smith 1992, Richardson 1995, Smith et al 2002) and recognise two subgenera. Support for subgenera within *Bothriembryon* will be examined in a near-comprehensive molecular systematic assessment of the genus (Kirkendale et al. in prep.).

#### Bothriembryon (Bothriembryon) sophiarum sp. n.

Taxon classificationAnimaliaStylommatophoraBothriembryontidae

http://zoobank.org/2EE13185-B302-42DC-9E55-DAFAB0B44899

**Figure 1.**
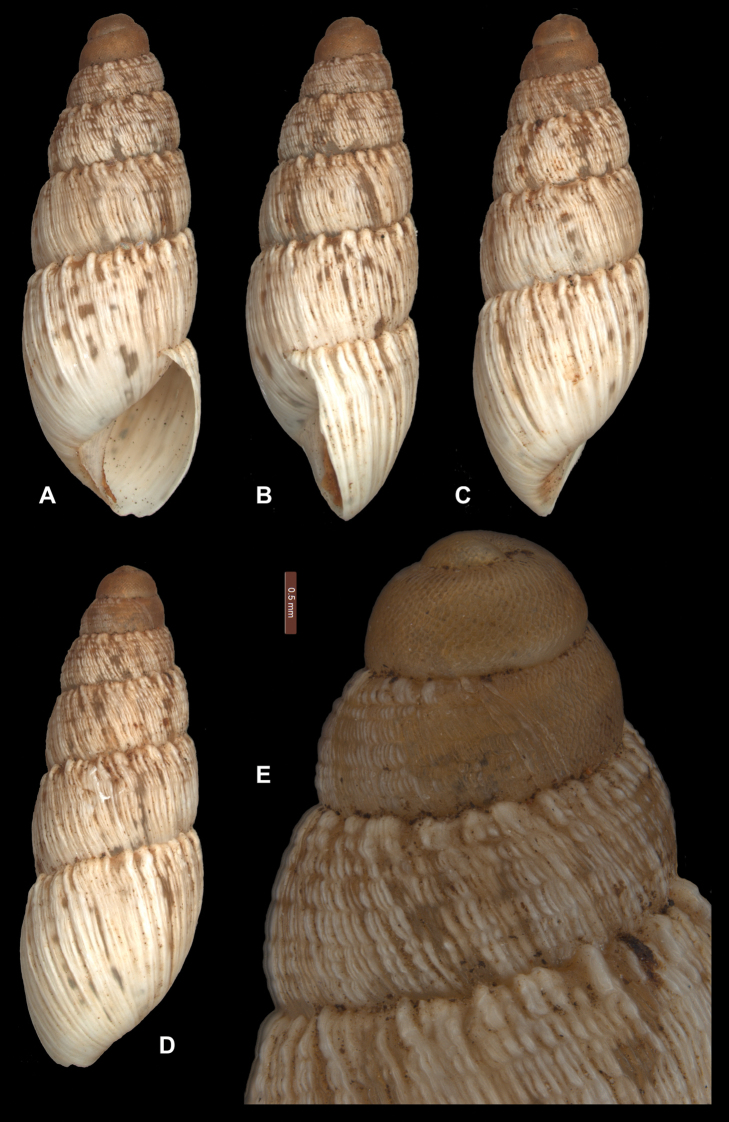
Bothriembryon (Bothriembryon) sophiarum sp. n. **A–D** holotype WAM S66478 (H = 14.4 mm) **E** Protoconch and early teleoconch sculpture; scale line 0.5 mm.

Figs 1 and 3 , 4 , 5 , Table 1

##### Type material.

**Holotype.** Western Australia, Nullabor Plain, Baxter Cliffs near Burnabbie Ruins, 32°07'30"S, 126°20'45"E, V. Kessner collector (ex J. Hemmen collection) 6 October 1989, dry, WAM S66478. **Paratypes** (from type locality) WAM S66479 (2 dry specimens) and RMNH.334653 (2 dry specimens); Western Australia, Nullabor Plain, Baxter Cliffs near Burnabbie Ruins, 32°07'30"S, 126°20'45"E, V. Kessner collector, 6 October 1989, WAM S30768 (6 dry specimens), AM C.477954 (3 dry specimens), RMNH.334654 (1 dry specimen).

##### Other material examined.

Western Australia: Israelite Bay, W.G. Buick Collection No. 13096, Pre June 1992, WAM S7972 (2 dry specimens); Israelite Bay area, start of cliffs at E end, Point Culver area, A. Longbottom, 21 October 1983, WAM S7977 (46 dry specimens); Nuytsland Nature Reserve, Toolina Cove, A. Cummings, 31 August.2010, WAM S64829 (4 preserved specimens); Top of Toolina Cliff, J. Lowry, 07 November 1966, WAM S7978 (8 dry specimens); Nuytsland Nature Reserve, Baxter Cliffs, near Baxter Memorial, A. Cummings, 30 August 2010, WAM S64824 (6 preserved specimens); South of Baxter Memorial, 50 feet from edge of cliff, P. Bridge and B. Robinson, 19 December 1966, WAM S7968 (6 dry, 24 preserved specimens); 38 km S of Caiguna, sea cliff top, K.A. Lance, 07 January 1976, WAM S7966 (25 dry specimens); S of Caiguna, between the Baxter Memorial and coast, P. Bridge and B. Robinson, 19 December 1966, WAM S7971 (8 dry specimens); S of Caiguna; near coast, B. Robinson, April 1966, WAM S8006 (2 dry specimens); 6 km SE of Baxter Memorial, top of Baxter Cliffs, A. Saar and K. Lance, 07 January 1976, WAM S7970 (10 dry, 4 preserved specimens); Twilight Cove, on cliff slope east of the cave, J. Lowry, 05 November 1966, WAM S7973 (33 dry specimens); 13 miles SE of Cocklebiddy, 7 miles N of Eyre, K. Thies, 21 May 1971, WAM S7974 (12 dry specimens); Eyre Homestead, escarpment face, W. Humphreys, March 1985, WAM S7969 (1 dry specimen); 14 miles ESE of Cocklebiddy, on face of Hampton Escarpment, A. Baynes and W. Youngsen, 04 September 1969, WAM S8031 (25 dry, 2 preserved specimens); Eyre, foot of escarpment, E. Sedgwick, August 1977, WAM S8053 (8 dry specimens); Baxter Cliffs 1.3 km E of Burnabbie Ruins, 32°07'6.54"S, 126°21'4.50"E, R. Phillips, 6 March 2015, WAM S67680 (1 wet specimen).

##### Diagnosis.

A slender shell characterised by plicate teleoconch whorls, often with pillared sculpture formed from incised spiral lines which become less frequent on the body whorl, and a strongly crenulate suture.

##### Description.

*Shell morphology*. Shell slender, mostly turriform, diameter 4.7–6.7 mm (mean 5.5 mm, sd 0.45), height 12.7–24.4 mm (mean 16.2 mm, sd 2.39) with 6.20–8.50 whorls (mean 7.05, sd 0.63) and a H/D ratio of 2.4–3.8 (mean 2.9, sd 0.26), rimate (Table 1, Suppl. material 1). Protoconch of 1.80–2.45 whorls (mean 2.18, sd 0.14) with very short, separate oblique wrinkles extending from suture before reticulating into a dense pattern of uniform punctated thimbles (honeycomb pattern). Teleoconch consisting of slightly convex, but regularly rounded plicate whorls, sculptured with narrow, crowded (often bifurcate) flattened or slightly raised axial ribs that are smooth and often translucent. The axial ribs become irregularly spaced on the last whorl, fading away towards the lower part of the whorl. Axial ribs usually crossed by only a few (mean 5.0, sd 1.0 on penultimate whorl) faint incised spiral lines creating a pillared sculpture that becomes less obvious on the body whorl. Suture irregularly but strongly crenulate formed from axial ribs terminating as large, rectangular nodules at the suture line, with a single nodule often forming from multiple axial ribs. Colour reddish-brown at the protoconch, the teleoconch cream with irregular blotches of reddish- to greyish-brown. Aperture relatively small, skewed elongate-ovate, lip thin, simple, basal margin slightly angular at the transition to the columellar margin, which is triangular dilated above; parietal callus thin and transparent.

**Table 1.** Shell measurements of the type material of Bothriembryon (Bothriembryon) sophiarum sp. n.

Bothriembryon (Bothriembryon) sophiarum Registration number	n	Shell height mm (sd)	Shell diameter mm (sd)	H/D Ratio (sd)	No. Whorls (range)
WAM S66478 (Holotype)	1	14.4	5.0	2.9	6.75
WAM S66479	2	12.7 (0)	4.7 (0)	2.7 (0)	6.28 (6.20–6.35)
RMNH.334653	2	13.7 (0.35)	5.0 (0)	2.7 (0.07)	6.48 (6.20–6.75)
WAM S30768	6	15.3 (1.07)	5.4 (0.30)	2.8 (0.07)	6.62 (6.25–7.00)
AM C.477954	3	15.2 (0.06)	5.4 (0.21)	2.8 (0.12)	6.57 (6.50–6.60)
RMNH.334654	1	16.2	5.7	2.8	6.85
**Grand Mean**	**15**	**14.7 (1.23)**	**5.2 (0.35)**	**2.8 (0.09)**	**6.56 (6.20–7.00)**

*Animal external morphology*. Body and foot sculptured with regular honeycomb pattern. Upper body and tentacles dark brown to black with an olive to green foot base and sides, the latter relatively wide (Fig. 3A).

**Figure 2.**
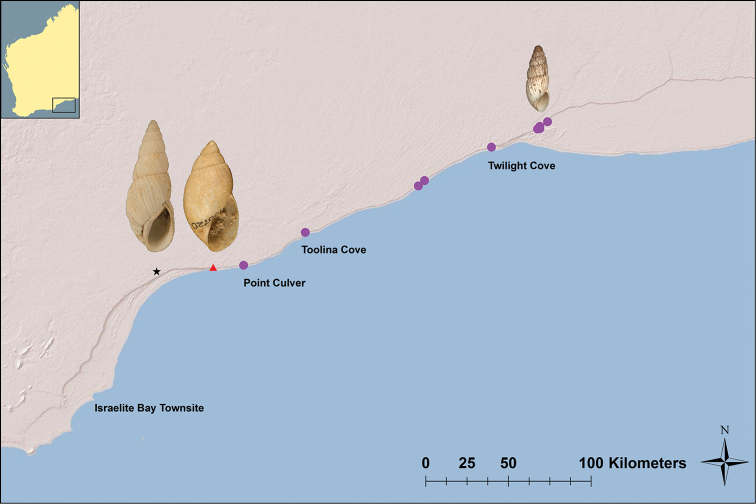
Distribution map of Bothriembryon (Bothriembryon) sophiarum sp. n. (dot) including photo of holotype (WAM S66478, H = 14.4 mm) and type localities and photos of nearby coastal species Bothriembryon (Bothriembryon) perditus Iredale, 1939 (triangle, AM C100720, H = 23.2 mm) and Bothriembryon (Bothriembryon) gratwicki (Cox, 1899) (star, AM C127559, H = 29.5 mm); Inset: Western Australia highlighting enlarged area

**Figure 3.**
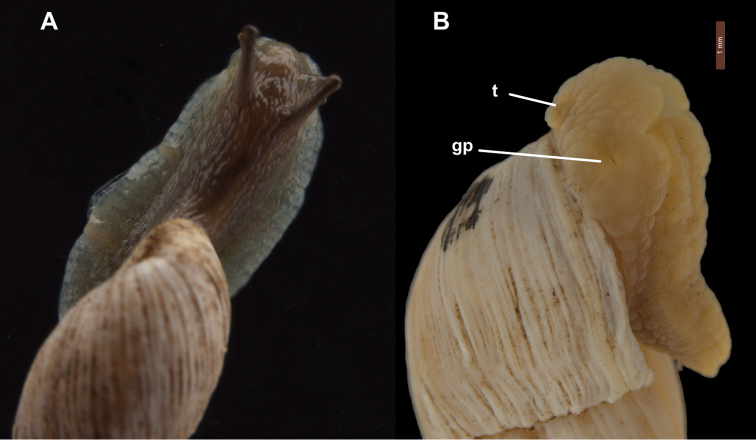
**A** Bothriembryon (Bothriembryon) sophiarum sp. n., **A** living animal WAM S67680 **B** relaxed narcotized specimen showing the genital pore, WAM S7968. Scale lines 1.0 mm (B). Abbreviations: gp, genital pore; t, retracted tentacle.

**Figure 4.**
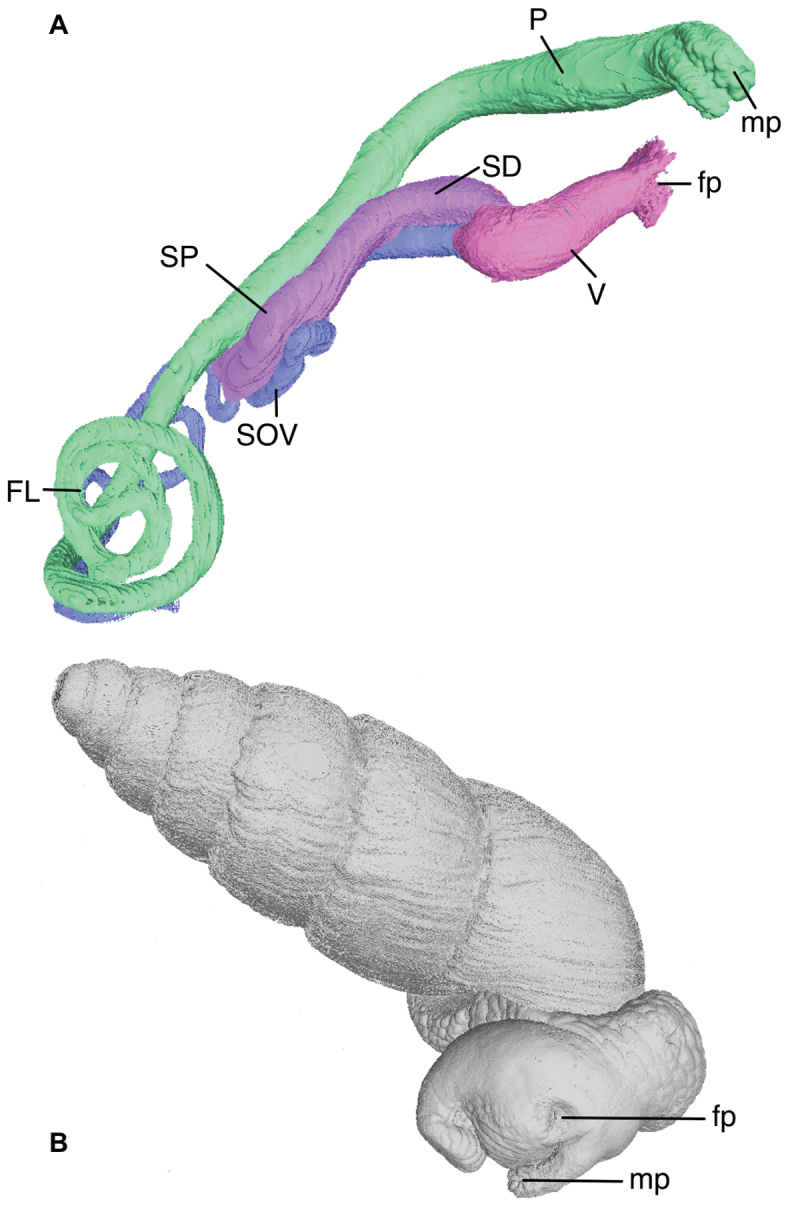
Bothriembryon (Bothriembryon) sophiarum sp. n., WAM S7968, genitalia (extruded) **A** segmented in Mimics to show the different parts **B** *in situ*. Abbreviations: FL, flagellum; fp, female pore; mp, male pore; P, penis (or phallus); SD, spermathecal duct; SOV, spermoviduct; SP, spermatheca (or bursa copulatrix); V, vagina.

**Figure 5.**
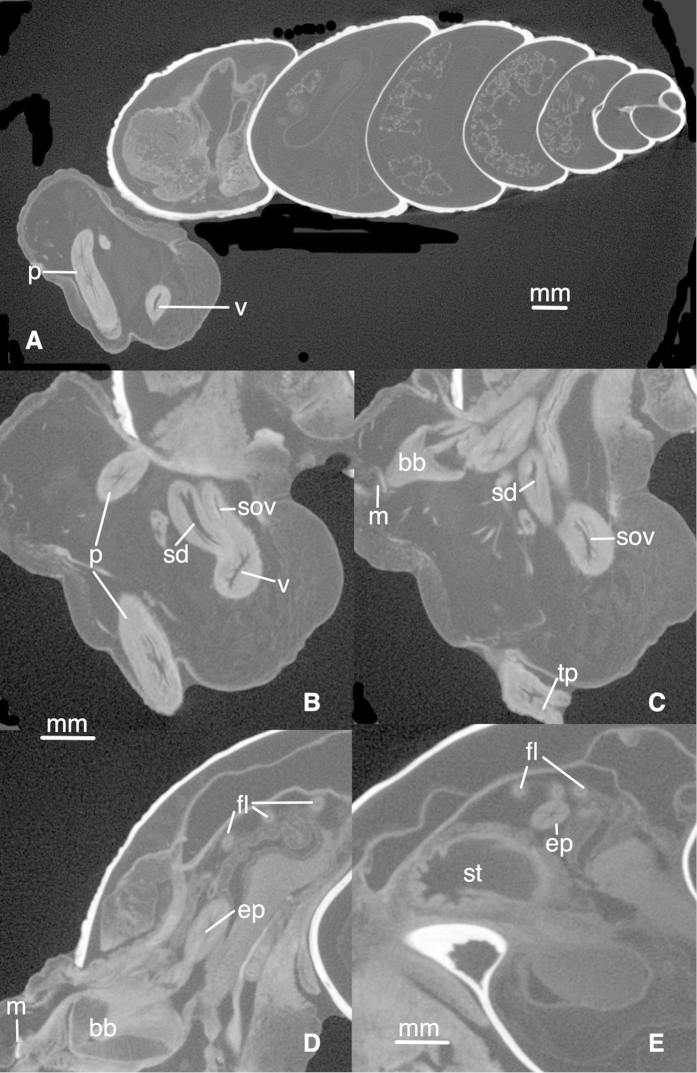
Bothriembryon (Bothriembryon) sophiarum sp. n., WAM S7968, anatomy shown with micro-CT. **A** Longitudinal view of total snail **B–E** Details showing parts of genitalia at different cross-sections. Abbreviations: bb, buccal bulb; ep, epiphallus; fl, flagellum; m, mandibula; p, penis; sd, spermathecal duct; sov, spermoviduct; st, stomach; tp, tip of penis; v, vagina.

*Genital morphology*. (Based on micro-CT images, see Figs 4A–B, 5A–E) Phallus gradually becoming narrower, with the distal part of the epiphallus and the proximal part of the flagellum subcylindrical. Distal part of penis lumen star-shaped (five-legged), lined with a high epithelium and gradually changing into the epiphallus, of which the narrow lumen is also star-shaped. Near the transition to the flagellum the lumen becomes three-legged star-shaped with five very narrow side-branches; more proximally the lumen is rectangular with five very narrow side-branches. The vagina is externally swollen, internally the lumen is elongated and undivided in its distal part, becoming forked at the tail-ends near the split into the spermathecal duct and spermoviduct. The spermathecal duct is comparatively broad with a club-shaped bursa copulatrix. The spermoviduct is slender (as far as traceable). In 3D (Fig. 4A) the genitalia are extruded outside the body of the animal; the female part cannot be traced towards its distal end, the phallus is heavily curled towards its distal end.

##### Distribution.

Western Australia; along the escarpment and cliff tops of the Baxter Cliffs and Hampton Ranges from the Point Culver area eastward to the Burnabbie Ruins, a linear distance of about 180 kilometres (Fig. 2). Museum records (WAM S7972) suggest it might occur further westward to Israelite Bay (townsite) but the veracity of the location data is questionable.

##### Habitat.

**Figure 6.**
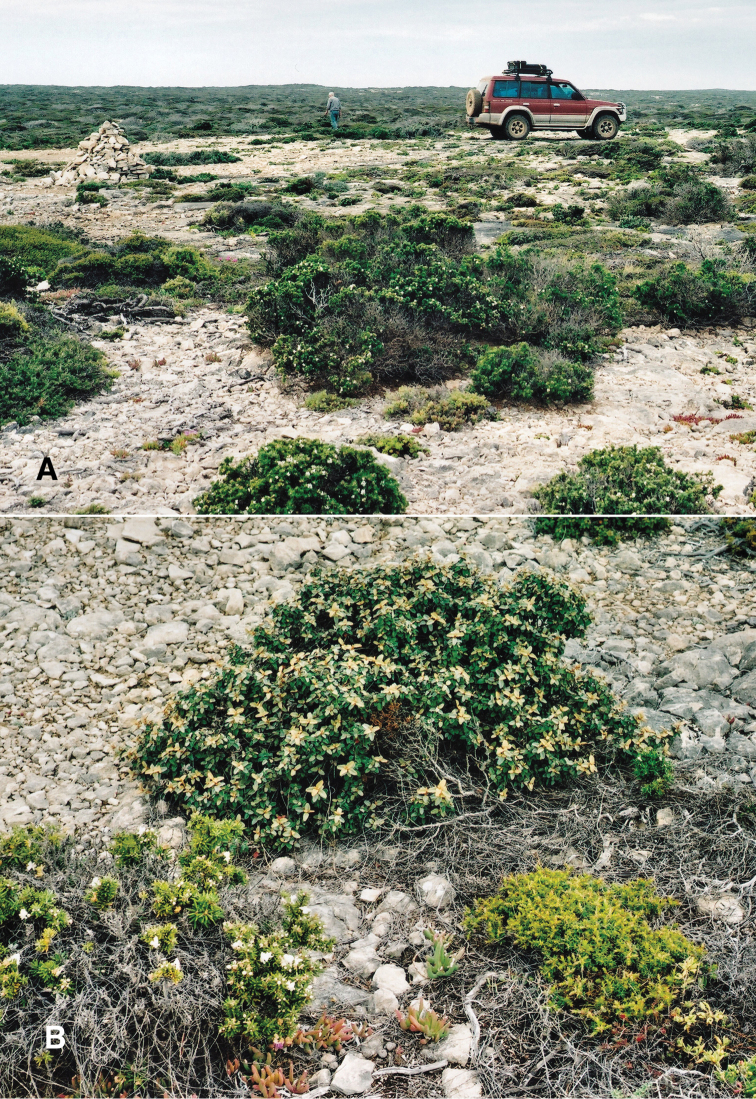
Bothriembryon (Bothriembryon) sophiarum sp. n., habitat for WAM S64824 **A** Inland view **B** Detail showing host plants *Westringia dampieri*, *Carpobrotus virens* and Correa backhouseana var. coriacea among limestone (photos courtesy Andrew Cummings and Ben Schneider).

Very open, low coastal scrub on limestone cliff-edge or slope scattered (often densely) with low limestone rocks and stones. Dominant plant species were *Westringia dampieri*, Correa backhouseana var. coriacea and *Carpobrotus virens* and very occasionally *Melaleuca* and *Eucalyptus* trees. In dry conditions living specimens are commonly found in rock crevices or fissures; under stones or around tree roots, and occasionally in litter. When wet, crawling snails have been observed on soil and stones and on branches of scrub (Fig. 6).

##### Remarks.

Bothriembryon (Bothriembryon) sophiarum can be distinguished from most other *Bothriembryon* species by its shell morphology, notably its slender turriform shape and a teleoconch sculpture of coarsely plicate whorls and strongly crenulate sutures. Most *Bothriembryon* species are ovate to elongate-conical in shape and have a teleoconch sculpture of faint or narrow axial growth lines. The nearby Bothriembryon (Bothriembryon) perditus Iredale, 1939 has similar shell morphology but its shell is much broader being elongate-conical in shape and has sutures which are more finely crenulate. The other nearby species Bothriembryon (Bothriembryon) gratwicki (Cox, 1899) is similar in shape but its shell is broader and usually more elongated, with a coarse nodulose teleoconch sculpture (Fig. 2). Only one fossil species occurs nearby, *Bothriembryon kremnobates* Kendrick, 2005 which is found further east on the Roe Plain and is ovate-conical in shape. Anatomically Bothriembryon (Bothriembryon) sophiarum differs only slightly from known Bothriembryon (Bothriembryon) species which have a long, narrow spermathecal duct and a short rounded bursa copulatrix. The short and broad spermathecal duct and relatively broad, elongate bursa copulatrix of Bothriembryon (Bothriembryon) sophiarum agrees more with Bothriembryon (Tasmanembryon) tasmanicus (Kershaw 1986, Breure 1978). However in this paper we have tentatively placed *Bothriembryon sophiarum* in the subgenus *Bothriembryon* on account of its shell morphology and its geographical proximity to other members of the same subgenus. The specimens examined have their genitalia somewhat extruded, hence the male and female genital pores seem to be separated; in other preserved, non-extruded specimens these pores are united inside the atrium (Fig. 4A). It is interesting to note that Bothriembryon (Bothriembryon) sophiarum specimens from Point Culver (WAM S7977) at the western edge of its range, are slightly taller (mean height 20.3 mm, sd 2.11) with a higher H/D ratio (mean 3.4). This collection is a large series (n = 46) and most likely represents population variation due to local environmental conditions, a common occurrence within *Bothriembryon* as suggested by Main and Carrigy (1953).

##### Etymology.

Named in honour of Sophie Jade Whisson, first daughter of the senior author and Sophie J. Breure, spouse of the second author; noun in plural genitive case.

## Discussion

Bothriembryon (Bothriembryon) sophiarum appears to have a restricted range with a linear distribution of ca. 180 km and as it currently occupies an area less than 10000 km^2^ qualifies as a Short Range Endemic (SRE) (Harvey 2002). Like many land snail species in arid environments (Slatyer et al. 2007) Bothriembryon (Bothriembryon) sophiarum has developed strategies to avoid desiccation, such as occupying a niche of rocky near coastal cliff-edges and escarpments, an environment that would support higher rainfall and lower temperatures as well as provide shelter and shade. Live Bothriembryon (Bothriembryon) sophiarum collected in the hot dry months suggests they mostly aestivate within rocks fissures or loosely under rocks on the soil surface, being a free-sealer with a white calcareous epiphragm for long-term aestivation. Resting specimens that were recently active have been observed with a clear mucoid seal over the aperture. This observed aestivation pattern of Bothriembryon (Bothriembryon) sophiarum fits the definition of a rock-dweller provided by Heller (1987), where rock-dwelling land snails aestivate during summer in rock crevices, cliffs, among boulders or beneath stones. This pattern also contains those species that burrow in soil but will rest temporarily beneath stones or between crevices of boulders

Bothriembryon (Bothriembryon) sophiarum has a slender, high-spired shell shape which is intriguing and differs from almost all known members of *Bothriembryon* which are predominantly ovate to elongate-conical in shape. Breure and Whisson (2012) remarked that the nearby and similarly shaped *Bothriembryon gratwicki* (Cox, 1899) was “aberrant within the genus *Bothriembryon*” (see Fig. 2). Heller (1987) found that there were differences in shell shape depending on what habitat a species occupied and that shell form was largely governed by the foot size requirements for each habitat and the ability to move easily within a habitat. The rocky limestone substrate in which Bothriembryon (Bothriembryon) sophiarum is found is often fractured with narrow cracks and fissures, and the slender shell shape would allow easy access into these cavities and/or under rocks. It would also aid climbing the vertical surface of the rocks. Additionally, the shell colour of Bothriembryon (Bothriembryon) sophiarum (cream with red/grey brown blotches) may provide camouflage from predators while in or on the similarly coloured limestone rocks.

## Supplementary Material

XML Treatment for Bothriembryon

XML Treatment for Bothriembryon (Bothriembryon) sophiarum

## Supplementary materials

Supplementary material 1
Bothriembryon (Bothriembryon) sophiarum measurements of all material examined. Data type: specimens measurements Explanation note: See main text for abbreviations and methods used. This dataset is made available under the Open Database License (http://opendatacommons.org/licenses/odbl/1.0/). The Open Database License (ODbL) is a license agreement intended to allow users to freely share, modify, and use this Dataset while maintaining this same freedom for others, provided that the original source and author(s) are credited.Corey S. Whisson, Abraham S.H. Breure
